# A Case of Chronic Ectopic Pregnancy Manifested by Rectal Bleeding

**DOI:** 10.1155/2017/5974590

**Published:** 2017-05-14

**Authors:** Nina Vukas Radulovic, Maria Bullarbo, Erling Ekerhovd

**Affiliations:** Department of Obstetrics and Gynecology, Institute of Clinical Sciences, Sahlgrenska Academy, University of Gothenburg, Göteborg, Sweden

## Abstract

Ectopic pregnancy resulting in perforation of the rectum and rectal bleeding is clinically rare. We report an extremely rare case of chronic ectopic pregnancy with decreasing low levels of serum *β*-HCG resulting in rectal bleeding. A 31-year-old woman, gravida 3, para 3, with moderate abdominal pain and rectal bleeding was diagnosed with a tubal pregnancy. The tube was adherent to the rectum. Following salpingo-oophorectomy, the perforation of the rectum was sutured. Biopsies from the rectum as well as the tube confirmed chronic ectopic pregnancy. This case illustrates that diagnosing ectopic pregnancy is sometimes extremely challenging and it underlines the importance of follow-up consultations when the final diagnosis has not yet been reached.

## 1. Introduction 

Ectopic pregnancy is diagnosed in 1.3–2.0% of all reported pregnancies and is an important cause of maternal morbidity and occasionally mortality [1]. Symptoms and signs of ectopic pregnancy vary but include abdominal or pelvic pain, missed period, vaginal bleeding, and gastrointestinal or urinary symptoms as well as pressure or pain on defecation. Transvaginal sonography (TVS) is the method of choice for the diagnosis of ectopic pregnancy [2]. An ectopic pregnancy can frequently be visualized by TVS as an inhomogeneous pelvic mass and free fluid within the pouch of Douglas. In fact, the increased frequency of positive scans has led to a reduction in the number of unnecessary laparoscopies [3].

Ectopic pregnancies with low serum human chorionic gonadotrophin (hCG) levels are known to be especially challenging to diagnose. Chronic ectopic pregnancy is such a subgroup and is characterized by symptoms and signs of mild to moderate intensity and low serum levels of *β*-HCG [4, 5]. Chronic ectopic pregnancy resulting in perforation of the rectum and rectal bleeding despite moderate symptoms and decreasing low levels of *β*-HCG, as described in the present report, represents a novel clinical complication of this type of pregnancy.

## 2. Case Presentation 

A previously healthy 31-year-old woman, gravida 3, para 3, was admitted to the hospital with a history of lower abdominal pain lasting more than two weeks. On the day she was admitted, her pain had suddenly increased in intensity and she had noted the presence of fresh blood on her toilet paper.

The woman had been examined by a gynecologist three days earlier after experiencing mild to moderate abdominal pain. She had had a copper intrauterine device (IUD) for three years and usually had regular periods. However, for the last few weeks, she had only experienced some spotting. A physical examination by the gynecologist revealed some vaginal discharge, but no vaginal bleeding. The uterus and the adnexa were tender when bimanual palpation was performed. Ultrasound showed an endometrial lining of approximately 5 mm. The IUD was located in the uterine cavity. Both ovaries were normal, but some fluid was seen in the right fallopian tube. No fluid was visualized in the pouch of Douglas. The gynecologist tried to remove the IUD, but this procedure was unsuccessful since the IUD strings could not be seen. The patient's temperature was 36.7°C. However, routine biochemistry showed that serum CRP was 100 g/L and *β*-HCG was 412 mIU/mL. The gynecologist concluded that the woman had a pelvic infection and prescribed doxycycline and metronidazole tablets. As the serum *β*-HCG was suggestive of an early stage of pregnancy, an ectopic pregnancy could not be excluded. A follow-up consultation was therefore scheduled for three days later. The patient was also encouraged to contact a hospital immediately if her condition worsened.

Two days later, the patient saw fresh blood on her toilet paper. Her pain was also more intense. Following the recommendation of the gynecologist, she contacted the hospital. When she arrived at the hospital, her condition was in general good. She reported pain of mild to moderate intensity. No blood was seen in the vagina, but gynecological examination revealed a fixed tender mass behind her uterus. The size of the mass was difficult to assess due to patient tenderness. However, TVS confirmed a 6 × 5 cm mass containing hypoechoic and anechoic areas. Very little fluid was visualized in the pouch of Douglas. Rectal examination revealed fresh blood. Due to rectal bleeding, a rectoscopy was performed, which identified a 1.5 cm wide perforation in the anterior wall of the rectum surrounded by tumor-like tissue with active bleeding about 15 cm from the anal verge. Several biopsies were obtained for histopathological examination since malignancy could not be excluded. Laboratory tests showed that serum CRP had increased to 179 g/L, while *β*-HCG had decreased to 366 mIU/mL. The patient's hemoglobin count was 11.5 g/100 mL.

A laparoscopy was performed immediately after the rectoscopy. Several pelvic inflammatory bowel adhesions had to be removed before the right fallopian tube was identified in the pouch of Douglas. The ampullary part of the fallopian tube was dilated to approximately 5 cm. Both the tube and the right ovary were substantially damaged due to inflammatory reaction and a salpingo-oophorectomy was performed. The rectal perforation was identified and sutured. The patient's postoperative recovery was uneventful and serum *β*-HCG was undetectable five days after laparoscopy.

**Figure 1:**
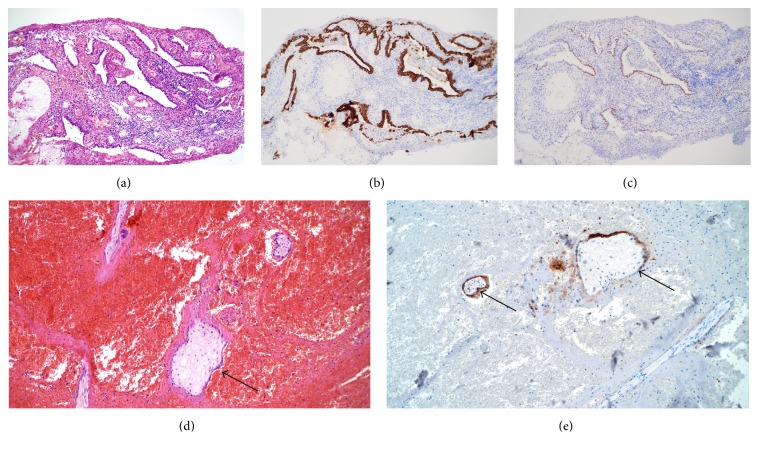
Biopsies from the rectal wall with glandular epithelium (a) with positive staining for CK7 (b) and estrogen receptor (c) indicating an origin from the gynecological tract. Biopsies from the removed fallopian tube with chorion villi ((d), arrow) with positive staining for HCG ((e), arrow).

Cervical and vaginal swabs for bacterial culture taken at the initial gynecological examination were negative. Histopathological examination of biopsies from both the rectum and the tube confirmed chronic ectopic pregnancy (Figure 1). Chronic and acute inflammatory reactions were diagnosed in tissue biopsies from the fallopian tube and the removed ovary as well as in rectal biopsies. There were no signs of malignancy.

## 3. Discussion 

Rectal bleeding is an extremely rare complication of ectopic pregnancy. A review of the literature found only eight cases, all representing acute and dramatic events involving massive intestinal bleeding caused by interstitial tubal pregnancies or true abdominal pregnancies [6]. The terminal ileum, sigmoid colon, and caecum were the parts of the gastrointestinal tract most commonly involved. The rectum was the source of bleeding in only one case.

In the present report, the symptoms were of mild to moderate intensity and had been ongoing for more than 2 weeks. Rectal bleeding was also modest. As confirmed by the histopathological examination, the patient suffered from a chronic ectopic pregnancy. This type of pregnancy accounts for approximately 6% of all ectopic pregnancies and typically presents with repeated episodes of lower abdominal pain and vaginal bleeding of varying severity [7]. Although the pathogenesis of chronic ectopic pregnancy is not fully known, it has been hypothesized that growth of trophoblastic tissue causes gradual destruction of the tubal wall, resulting in a slow, episodic leakage of blood [4]. The presence of blood, trophoblastic tissue, and disrupted tubal tissue in the peritoneal cavity is thought to provoke an inflammatory reaction resulting in the development of an organized hematocele and adhesions.

Chronic ectopic pregnancy is often difficult to differentiate from pelvic inflammatory disease, endometriosis, and uterine fibroids when TVS is performed [7]. The mass that occurs in this type of pregnancy is typically produced by adhesions between the inflamed tube and surrounding structures, blood, and necrotic debris after degeneration of the conceptus, yielding a heterogenous echo pattern [8].

In the present case, fluid was seen in the right fallopian tube at the initial examination. The ultrasound finding combined with a serum CRP of 100 g/L indicated that the patient suffered from pelvic inflammatory disease. Antibiotics were therefore prescribed. However, cervical and vaginal swabs taken at the initial gynecological examination were negative. Thus, the high CRP levels probably reflect that the pelvic inflammation had reached a stage where the gastrointestinal tract had become involved. The prescription of antibiotics at the initial consultation was clearly due to a misdiagnosis and can only be described as mismanagement.

An intrauterine pregnancy can be visualized using TVS with serum *β*-HCG levels as low as 1000 IU/L [9]. In cases of chronic ectopic pregnancy, serum *β*-HCG levels are usually much lower due to a very small amount of live villi [10]. It is of immense clinical importance that follow-up consultations are performed until the final pregnancy outcome is known. Serial serum *β*-HCG measurements are clearly helpful in cases where an ectopic pregnancy cannot be excluded. However, in some cases of chronic ectopic pregnancy, serum *β*-HCG levels are undetectable and pregnancy tests are therefore negative [10, 11].

The present case is unique because it shows that chronic ectopic pregnancy can cause perforation of the rectum, despite symptoms of moderate intensity and decreasing low levels of serum *β*-HCG.
